# Exploring potential polysaccharide utilization loci involved in the degradation of typical marine seaweed polysaccharides by *Bacteroides thetaiotaomicron*

**DOI:** 10.3389/fmicb.2024.1332105

**Published:** 2024-05-09

**Authors:** Biao Yu, Zheng Lu, Saiyi Zhong, Kit-Leong Cheong

**Affiliations:** ^1^Guangdong Provincial Key Laboratory of Aquatic Product Processing and Safety, Guangdong Province Engineering Laboratory for Marine Biological Products, Guangdong Provincial Engineering Technology Research Center of Seafood, Guangdong Provincial Science and Technology Innovation Center for Subtropical Fruit and Vegetable Processing, College of Food Science and Technology, Guangdong Ocean University, Zhanjiang, China; ^2^Department of Biology, College of Science, Shantou University, Shantou, China; ^3^School of Life and Health Sciences, Hainan University, Haikou, China

**Keywords:** *Bacteroides thetaiotaomicron*, marine algae polysaccharides, CAZymes, polysaccharide utilization loci, polysaccharide metabolism

## Abstract

**Introduction:**

Research on the mechanism of marine polysaccharide utilization by *Bacteroides thetaiotaomicron* has drawn substantial attention in recent years. Derived from marine algae, the marine algae polysaccharides could serve as prebiotics to facilitate intestinal microecological balance and alleviate colonic diseases. *Bacteroides thetaiotaomicron*, considered the most efficient degrader of polysaccharides, relates to its capacity to degrade an extensive spectrum of complex polysaccharides. Polysaccharide utilization loci (PULs), a specialized organization of a collection of genes-encoded enzymes engaged in the breakdown and utilization of polysaccharides, make it possible for *Bacteroides thetaiotaomicron* to metabolize various polysaccharides. However, there is still a paucity of comprehensive studies on the procedure of polysaccharide degradation by *Bacteroides thetaiotaomicron*.

**Methods:**

In the current study, the degradation of four kinds of marine algae polysaccharides, including sodium alginate, fucoidan, laminarin, and Pyropia haitanensis polysaccharides, and the underlying mechanism by *Bacteroides thetaiotaomicron* G4 were investigated. Pure culture of *Bacteroides thetaiotaomicron* G4 in a substrate supplemented with these polysaccharides were performed. The change of OD600, total carbohydrate contents, and molecular weight during this fermentation were determined. Genomic sequencing and bioinformatic analysis were further performed to elucidate the mechanisms involved. Specifically, Gene Ontology (GO) annotation, Clusters of Orthologous Groups (COG) annotation, and Kyoto Encyclopedia of Genes and Genomes (KEGG) pathway enrichment were utilized to identify potential target genes and pathways.

**Results:**

Underlying target genes and pathways were recognized by employing bioinformatic analysis. Several PULs were found that are anticipated to participate in the breakdown of these four polysaccharides. These findings may help to understand the interactions between these marine seaweed polysaccharides and gut microorganisms.

**Discussion:**

The elucidation of polysaccharide degradation mechanisms by *Bacteroides thetaiotaomicron* provides valuable insights into the utilization of marine polysaccharides as prebiotics and their potential impact on gut health. Further studies are warranted to explore the specific roles of individual PULs and their contributions to polysaccharide metabolism in the gut microbiota.

## Introduction

Marine algae polysaccharides are complex carbohydrates extracted from a variety of marine seaweed. And they can be classified into several categories regarding their resources, structure, chemical components, and so on (Patel et al., 2023). A broad range of polysaccharides can be identified in seaweed, including sodium alginate, fucoidan, laminarin, and so on. Each class possesses particular characteristics and applications (Grundy et al., 2016; Zheng et al., 2020). Because of the special living condition of the algae, the synthesizing process of marine algae polysaccharides distantly differs from that of the terrestrial plant, for which the algae generate many active polysaccharides with novel structures and unique characteristics (Zheng et al., 2020; Xie and Cheong, 2022). Marine algae polysaccharides have garnered significant interest these years because of their biological functions in food manufacturing and pharmaceutical applications (de Jesus Raposo et al., 2015; Ebrahimzadeh et al., 2023). They might produce various positive impacts, such as anti-inflammatory, anti-virus, anti-cancer, immunomodulatory, food packaging, drug delivery, and hypoglycemic properties (Shannon and Abu-Ghannam, 2016; Zhao et al., 2018; Cheong et al., 2022; Jayakody et al., 2022; Obluchinskaya et al., 2022; Yao et al., 2022; Zhang et al., 2022).

The human gut microbiota (HGM), often referred to as the gut microbiome, comprises a diverse assembly of microorganisms that populates the digestive tract (Eckburg et al., 2005; Gomaa, 2020). These microorganisms include bacteria, fungi, and other archaea. In previous studies, it has been revealed that HGM is associated with the progression of various health disorders, including inflammatory bowel disease, anti-aging, obesity, diabetes, Parkinson’s disease, Alzheimer’s disease, asthma, and even alters how the host responds to drugs in the treatment of diseases (Ley et al., 2005; Arrieta et al., 2015; Hu et al., 2016; Leiva Gea et al., 2018; Bárcena et al., 2019; Khan et al., 2019; Sarkar and Banerjee, 2019; Contarino et al., 2023). The HGM contains the largest collection of microbes, which are embedded with millions of genes that render these hundreds of kinds of bacterial species with genes missing in our human genome. The encoded genes in the microbiota provide them with the capacity to metabolize the indigestible polysaccharides (Holscher, 2017). Still, due to insolubility or a lack of hydrolytic enzymes encoded by humans, many complex polysaccharides consumed by humans are resistant to host-mediated breakdown (Xu et al., 2020; Li et al., 2023). These carbohydrates undergo minimal degradation and assimilation in the upper gastrointestinal tract, while they function as an essential resource of nutrients and energy for the microbiota in the distal gut (Sonnenburg et al., 2010; Ye et al., 2021). Simultaneously, the absorption of carbohydrates can modulate the abundance and diversity of intestinal microbiota, making them a potential tool for treating disorders by regulating HGM (Adak and Khan, 2019; Vaughn et al., 2019).

*Bacteroides* predominate the gut microbial community and comprise roughly 48% of the HGM. Besides, the *Bacteroides* possess far more carbohydrate-active enzymes (CAZymes) than any other sequenced bacterium, making them efficient polysaccharide degraders and excellent intestine settlers (El Kaoutari et al., 2013; Lapébie et al., 2019). *Bacteroides* are able to quickly adapt their strategies for consuming carbohydrates to fit our various omnivorous diets. The complex nutrition-microbiota-host interaction is significantly shaped by this crucial adaptability (Lin and Zhang, 2017; Cheong et al., 2022). Additionally, *Bacteroides*’ considerable capacity for using polysaccharides can provide appropriate glycans for other bacterial species, promoting harmonious coexistence within intricate intestinal communities (Lapébie et al., 2019). Moreover, the Bacteroidetes show a variety of beneficial functions, including boosting host immunity, maintaining intestinal microecological balance, promoting the formation of intestinal mucosal blood vessels, and reducing intestinal inflammatory diseases (Martens et al., 2008; Romani et al., 2020). *Bacteroides thetaiotaomicron*, *Bacteroides ovatus*, and *Bacteroides uniformis* prove to be the dominant species of the Bacteroidetes. Among the Bacteroidetes, *B. thetaiotaomicron* colonizes the intestine widely and produces more CAZymes that degrade the polysaccharides into monosaccharides than other strains (Xu et al., 2003; Chaudet and Rose, 2016; Luis et al., 2017).

The CAZy^1^ is a specialized database designed for presenting and analyzing genomic, structural, and biochemical data related to carbohydrate-active enzymes (CAZymes). In the CAZy database, the CAZymes are divided into five categories: glycoside hydrolases (GHs), glycosyltransferases (GTs), polysaccharide lyases (PLs), carbohydrate esterases (CEs), auxiliary activities (AAs), and recognition (carbohydrate-binding module, CBM) (Park et al., 2010; Wardman et al., 2022). The degradation of the polysaccharide skeleton involves GHs and PLs in an essential way. Polysaccharide lyases break glycosidic bonds of polysaccharide in the manner of non-hydrolytic cleavage, while glycoside hydrolases break glycosidic bonds by hydrolysis and/or rearrangement (Henrissat, 1991; Davies and Henrissat, 1995; Lombard et al., 2010). Such multi-modular enzymes might or might not be made as a component of the CAZyme consortia contained within the known polysaccharide utilization loci (PULs). These PULs represent collections of genes that encode proteins with interconnected functions utilized for detecting, adhering, breaking down, and transporting specific polysaccharide (Grondin et al., 2017; Terrapon et al., 2018).

The diverse variety of GHs and PLs signify the enormous polysaccharide substrate of *B. thetaiotaomicron*. Thus, *B. thetaiotaomicron* was selected as model strain of human gut bacteria that could undergo genetic mutations for the examination of polysaccharide metabolism (Sonnenburg et al., 2006; Brown and Koropatkin, 2021). The process by which *B. thetaiotaomicron* utilizes complex carbohydrates has been well explored, relying on the abundance of sequencing data. *B. thetaiotaomicron*’s in-depth research on polysaccharide degradation provides an approach to understanding how other gut bacteria, particularly those also belonging to the genus *Bacteroides*, break down carbohydrates.

The impact of the interaction between polysaccharide and *B. thetaiotaomicron* on the host community has been gradually unrevealed these years. A wide range of metabolites produced by *B. thetaiotaomicron*, including short-chain fatty acids (SCFAs), vitamins, and other nutrients, have been associated with numerous health-benefiting functions. These metabolites exert their effects by influencing the gut microbiota, modulating immune responses, and contributing to the gut-brain axis (Xiao et al., 2021; Contarino et al., 2023).

In the current research, we will focus on the utilization of several marine algae polysaccharides, that is, sodium alginate, fucoidan, laminarin, and *Pyropia haitanensis* (Rhodophyta) polysaccharides, and their relationship with *B. thetaiotaomicron* G4. Besides, the genome analysis was employed to discover the genes with varying expression patterns in *B. thetaiotaomicron* G4. Genomic sequencing and bioinformatic analysis revealed potential target genes and pathways involved. Moreover, predicted PULs and CAZymes engaged in the breakdown of these four polysaccharides were depicted. These discoveries could contribute to a better comprehension of the interactions between marine algae polysaccharides and the microorganisms residing in the gut.

## Materials and methods

### Materials and chemicals

Laminarin (*Eisenia bicylis* source) (Phaeophyceae) was purchased from Tokyo Chemical Industry Co., Ltd. (Tokyo, Japan). Sodium alginate and fucoidan (*Undaria pinnatifida* source) (Phaeophyceae) were brought from Sigma-Aldrich (Saint Louis, United States). *P. haitanensis* polysaccharides (PHP) was prepared using a method previously reported by our research team (Qiu et al., 2020; Yu et al., 2023). In brief, the decolorized and deproteinized *P. haitanensis* powder was boiled in a water bath at 90°C for 2 h after adding 10 times volume distilled water (w/v). Centrifugation was used to extract the mixture’s supernatant, which was then combined with three liters of 95% ethanol. Following centrifugation for isolation, the precipitate was dissolved in water, frozen in a −20°C freezer, and subjected to freeze-drying to obtain the PHP. Standard dextrans with molecular weight from 4.66 kDa to 496 kDa were purchased from Aladdin (Shanghai, China). _D_-glucose, _L_-cysteine hydrochloride, hemin, vitamin K_1_ were procured from Solarbio Science & Technology (Beijing, China). Brain heart infusion (BHI) was brought from Hopebio (Qingdao, China). All other chemicals of analytical grade were acquired from XiLong Scientific Co., Ltd. (Shantou, China).

### Microbial strains and medium

The microbial strain-*B. thetaiotaomicron* GDMCC 1.1104 (*B. thetaiotaomicron* G4) was bought from Guangdong Microbial Culture Collection Center (Guangzhou, China). Brain heart infusion broth (BHI) suppled with _L_-cysteine hydrochloride (0.5 g/L) and resazurin (0.001 g/L) was used as an enrichment medium. Basal medium containing yeast extract (2.0 g/L), peptone water (2.0 g/L), sodium chloride (0.1 g/L), potassium phosphate dibasic (0.04 g/L), potassium phosphate monobasic (0.04 g/L), magnesium sulfate heptahydrate (0.01 g/L), calcium chloride hexahydrate (0.01 g/L), sodium bicarbonate (2.0 g/L), hemin (0.05 g/L), _L_-cysteine hydrochloride (0.5 g/L), resazurin (0.001), bile salt (0.5 g/L), tween 80 (2 mL/L), and vitamin K_1_ (10 μL/L) was prepared as the previous research reported (Zeybek et al., 2020; Li S. et al., 2021). For batch cultures, the fermentation medium was created based on the basal medium supplemented with 1% (w/v) sodium alginate, fucoidan, laminarin, and PHP added as a carbohydrate source. Prior to inoculation, the pH value of medium was set within the 6.8–7.0 range using a pH meter. After being boiled, the medium was transferred to a 50 mL anaerobic culture bottle and purged with N_2_. Using butyl rubber stoppers and screw caps, the bottles were tightly sealed and subsequently sterilized at 121°C for 15 min. After undergoing filter sterilization with a 0.22 μm filter, the autoclaved medium was supplemented with heat-sensitive hemin and vitamin K1, after which it was cooled to 60°C. The strain was cultured in an anaerobic incubator (10% H_2_, 10% CO_2_, 80% N_2_) at 37°C.

### Population structure and genetic diversity analysis

Identification of bacterial strain was achieved through 16S rRNA sequencing, and MEGA6.0 was used to create a systematic phylogenetic tree through the Neighbor-Joining (NJ) method (Tamura et al., 2013). The *B. thetaiotaomicron* G4 strain was subsequently employed for further cultivation, structural genomics analysis, functional annotation, CAZys prediction, and description of PULs.

### Evaluation of bacterial growth on four polysaccharides

The freeze-dried *B. thetaiotaomicron* G4 was rehydrated and spread in the enrichment medium. After 48 h cultivation and passage to fresh enrichment medium twice, the bacteria in optimal growth vitality conditions were obtained and stored for further research. After the third activation, the obtained supernatant was subjected to centrifugation at 12,000 rpm at 4°C for 15 min. The bacterial pellet was rinsed and then resuspended in phosphate buffered saline (PBS; pH = 7.2) twice. After discarding the supernatant, the pellet was collected for the following processing steps.

The organisms after the last enrichment culture were used as inocula (1% v/v) and passaged to fermentation medium supplemented with 1% polysaccharides. During the fermentation, the fermentation broths were collected and taken out of the anaerobic culture bottle for further analysis at several time points (0, 12, 24, 48, and 72 h). The turbidity at OD_600_ was measured by a spectrophotometer (Shimadzu, Kyoto, Japan) to characterize the growth condition of *B. thetaiotaomicron* G4. Every fermentation operation was carried out independently three times. The fermentation medium without added organisms was used as a parallel sample for optical measurement.

### Characterization of polysaccharide degradation

Total sugar contents were evaluated by the phenol-sulfuric acid method (DuBois et al., 1956), and its procedure is as follows: in short, centrifugation was employed on the fermented broths, and the supernatant was taken. After diluting the supernatant with appropriate volumes of distilled water, 0.2 mL of the distilled supernatant was added to 2 mL tubes containing 1 mL of sulfuric acid and 0.2 mL of 5% phenol (w/v). The solutions were thoroughly stirred, and the tubes were sealed with parafilm, followed by a 100°C water bath for 20 min. Then, the absorption at a wavelength of 490 nm was measured.

The standard glucose solutions were formulated through the dissolution of 100 mg of glucose in 1 L of distilled water. And the glucose solution was further diluted to the following concentrations: 0, 0.02, 0.04, 0.06, 0.08, and 0.1 mg/mL. And the sugar contents of glucose standards were also analyzed as per the procedure above. The standard curve was calculated from the absorption of glucose standards, with which the sugar contents of the fermentation samples were obtained. The measurement processes above were repeated three times.

### Molecular weight analysis of the polysaccharide of fermentation broths

The supernatant of fermentation broth collected above was frozen for 48 h in a control-rate freeze dryer at −45°C while the vacuum was decreased to 11.3 Pa. After drying, the residual sugar was rehydrated in distilled water to the same volume of 1 mL. The samples’ molecular weight was assessed through high performance liquid chromatography (HPLC) as our previous study described (Yu et al., 2023). The evaporative light scattering detector (ELSD) and TSK G5000 PWXL column (30 cm × 7.8 mm, ID, 10 μL, Tosoh Co., Ltd., Tokyo, Japan) were employed in the chromatographic process. The injection of 10 μL samples into the column was complemented by a mobile phase of ammonium acetate, flowing at a rate of 0.8 mL/min. Throughout the procedure, the detector and column was maintained at 35 ± 0.1°C. By assuming that the retention time was directly proportional to the logarithm of the molecular weight of standards (4.66, 12.6, 50.0, 63.3, 126, 496 kDa), the molecular weight of the samples was determined.

### Genomic DNA extraction and sequencing

The microbial DNA was obtained from the bacterial pellet by applying the TIANamp Bacteria DNA Kit (Tiangen Biotech, China) in accordance with the manufacturer’s instructions. Prior to constructing DNA library, the concentration and quality of extracted DNA were measured at 260/280 nm by NanoDrop2000 (Thermo Fisher Scientific, United States). The FEA Enzyme Mix (Tiangen Biotech, China) was utilized to break DNA samples that met the quality criteria into fragments ranging from 200 to 500 bp. Subsequently, these fragments were ligated with sequencing adaptors by DNA ligase (Sheng et al., 2021). Following adaptor ligation, the DNA fragments with attached adaptors are subjected to polymerase chain reaction (PCR) amplification. Sequencing libraries were generated with the TIANSeq Fast DNA Library Kit (Tiangen Biotech, China). The paired-end sequencing with a read length of 150 base pairs was conducted according to the manufacturer’s guidelines for whole genome sequencing, utilizing the Illumina HiSeq 2500 sequencer on the Illumina HiSeq 2500 platform (San Diego, CA, United States), achieving a 100-fold sequencing depth. After the completion of Illumina sequencing, the raw data undergo a series of data analysis operations, including quality control, statistical analysis, genome assessment, and *de novo* assembly (Akbar et al., 2021; Dang et al., 2022).

### CGView map of the *Bacteroides thetaiotaomicron* G4 genome

CGView (Circular Genome Viewer, http://wishart.biology.ualberta.ca/cgview/) is a tool designed for creating interactive circular genome maps (Stothard and Wishart, 2005; Barakat et al., 2011). The genome circular map was applied to obtain a complete depiction of genomic properties, such as the distribution of genes on the forward and reverse strands, gene functional categorization based on COG categories, GC content, and genomic islands (Stothard et al., 2019).

### Gene Ontology enrichment analysis

GO annotations are particularly valuable in enhancing our comprehension of the biological significance represented by the genes. Within the GO analysis, the proposed genes were examined in the GO database^2^ (Ashburner et al., 2000; Blake et al., 2013). The biological functions of the putative genes were established by substantial GO enrichment analysis. Protein function annotations containing three subtypes, including biological process (BP), molecular function (MF), and cellular component (CC) were assigned according to the GO database.

### Clusters of Orthologous Groups annotation

The Clusters of Orthologous Group (COG) database is available at http://www.ncbi.nlm.nih.gov/COG. The website includes the following key characteristics: comprehensive list of all COGs with links to individual COG websites, COGs arranged by functional category, and COGs grouped according to functional complexes and pathways (Tatusov, 2001). Besides, COG database comprises species from various major systematic evolutionary lineages, and it is built upon integrated protein sequence comparisons of previously sequenced species (Galperin et al., 2015). Conducting EggNOG database (evolutionary genealogy of genes: non-supervised Orthologous Groups, http://eggnog.embl.de/) comparisons enables functional annotation and categorization of predicted proteins (Jensen et al., 2008; Powell et al., 2012).

### Kyoto Encyclopedia of Genes and Genomes pathway enrichment analysis

KEGG is an open-access database^3^ of signal transduction pathways (Kanehisa et al., 2004). The candidate genes’ links to relevant biochemical metabolic and signal transduction pathways were determined through enrichment analysis (Kanehisa et al., 2006).

### Carbohydrate-active enzyme annotation and PUL prediction

Relying on sequence similarity, the genomic data of *B. thetaiotaomicron* (BT) was queried against the combined CAZymes database (see text footnote 1) by applying dbCAN2 software with default settings^4^ (Huang et al., 2018). The extensive CAZymes of BT were assigned and annotated into six major module/domain families and subfamilies, including GHs, GTs, PLs, etc. The *SusC*-like and *SusD*-like genes are commonly employed as signatures for the identification and assignment of PUL loci. TonB-dependent transporters, known as *SusC*-like proteins, exhibit a distinct linear domain sequence, whereas *SusD*-like proteins are defined by either a single or a pair of domains (Cantarel et al., 2009). Utilizing the annotation information from the Pfam database,^5^ we sought *SusC*/D gene pairs and their homologous genes among all genes.

Our predictions involved classifying tandem *SusC*/*D*-like pairs based on the existence of adjacent *SusC*-like genes (upstream) and *SusD*-like genes (downstream) located on the same DNA strand. The occurrence of duplications involving *SusC*-like or *SusD*-like genes was also taken into account. Some PUL regulatory genes, such as the hybrid two-component system (HTCS) and extra-cytoplasmic functioning family of *α* (ECF-*α*), were also added to the PUL display.

Additionally, CAZymes gene loci were identified to investigate potential PULs in the *B. thetaiotaomicron* G4 strain. We integrated this information with available data from the PULDB database^6^ and literature references pertaining to PULs specific to particular polysaccharide structures (Terrapon et al., 2015). This enabled us to compile a comprehensive prediction of PULs in the genome of this particular *B. thetaiotaomicron* strain targeting specific polysaccharides.

### Statistical analysis

The statistics were processed using GraphPad Prism 9. (GraphPad Software Inc., San Diego, CA). Data were analyzed by IBM SPSS Statistics ver. 25 (IBM Corporation, Armonk, NY, United States) using One-way ANOVA, and the significance level was determined at a *p* < 0.05 were considered significant.

## Results

### Comparative phylogenetic analysis

The 16S rRNA of *B. thetaiotaomicron* G4 was extracted and sequenced. Using 16S rRNA sequences, a phylogenetic tree for *B. thetaiotaomicron* G4 was established. Comparison of the acquired sequences with the sequences in the GenBank database using the NCBI BLAST software package revealed the greatest similarity of the genes of *B. thetaiotaomicron* G4 to those of members of the genus *Becteroides*.

**Figure 1:**
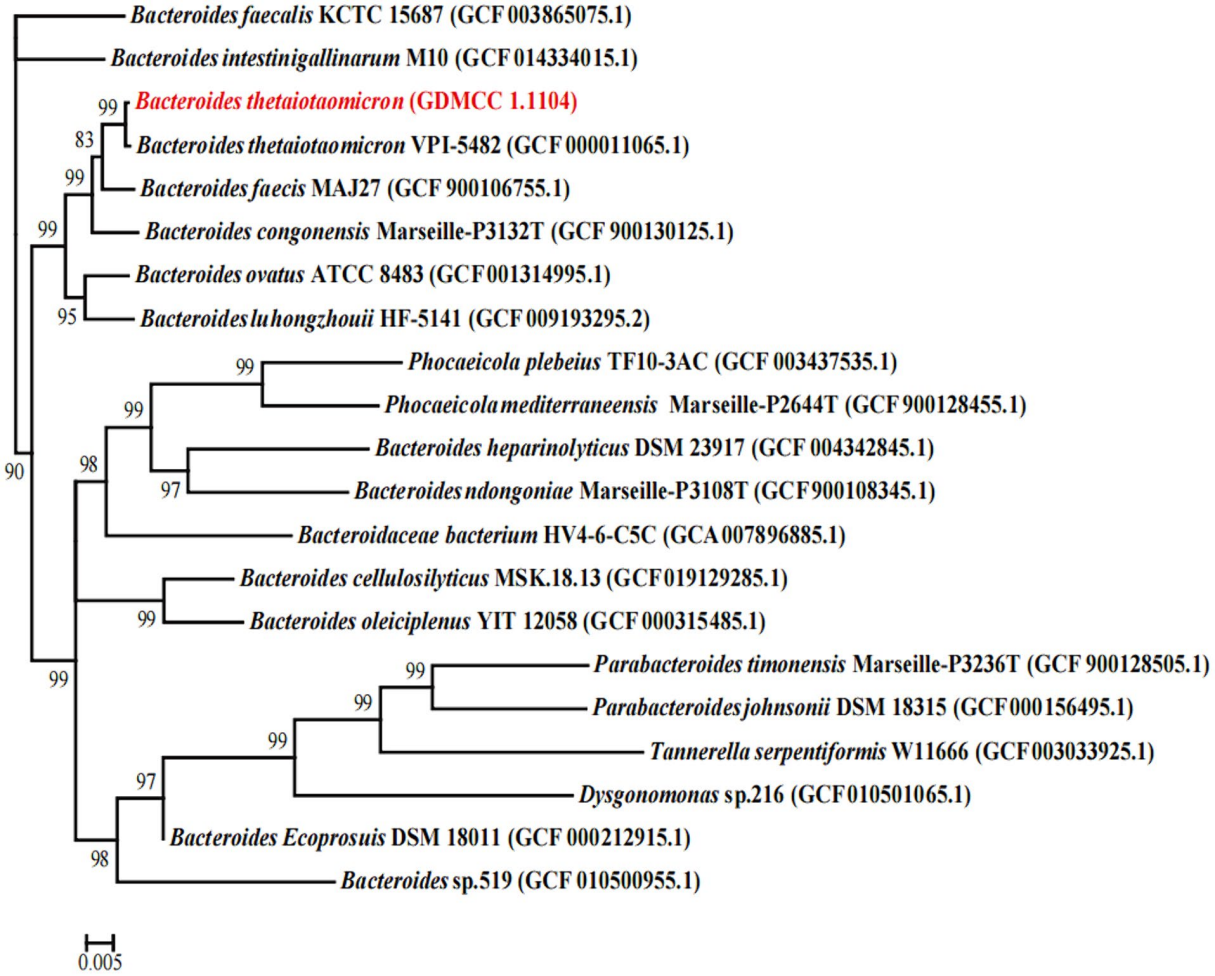
Phylogenetic tree showing the relatedness between the *Bacteroides thetaiotaomicron* G4, and the type strains of the genus *Becteroides*. The GenBank accession numbers of the sequences are given in parenthesis (www.ncbi.nlm.nih.gov). Bootstrap values (1,000 replicates) greater than 80% were shown. Numbers in parentheses are GenBank accession numbers. Scale bar, 0.005 substitution per nucleotide position.

In the phylogenetic analysis (Figure 1), *B. thetaiotaomicron* G4 and *B. thetaiotaomicron* VPI-5482 were clustered together closely with a bootstrap value of 99%, which indicated that they may possess the same origin. The comparative analysis of 16S rRNA between *B. thetaiotaomicron* G4 and *B. thetaiotaomicron* showed a similarity of 99.869%, which indicated that the BT strain used in the present study had a relatively high probability of belonging to *B. thetaiotaomicron*.

### The growth curve of *Bacteroides thetaiotaomicron* G4 on four typical marine algae polysaccharides

**Figure 2:**
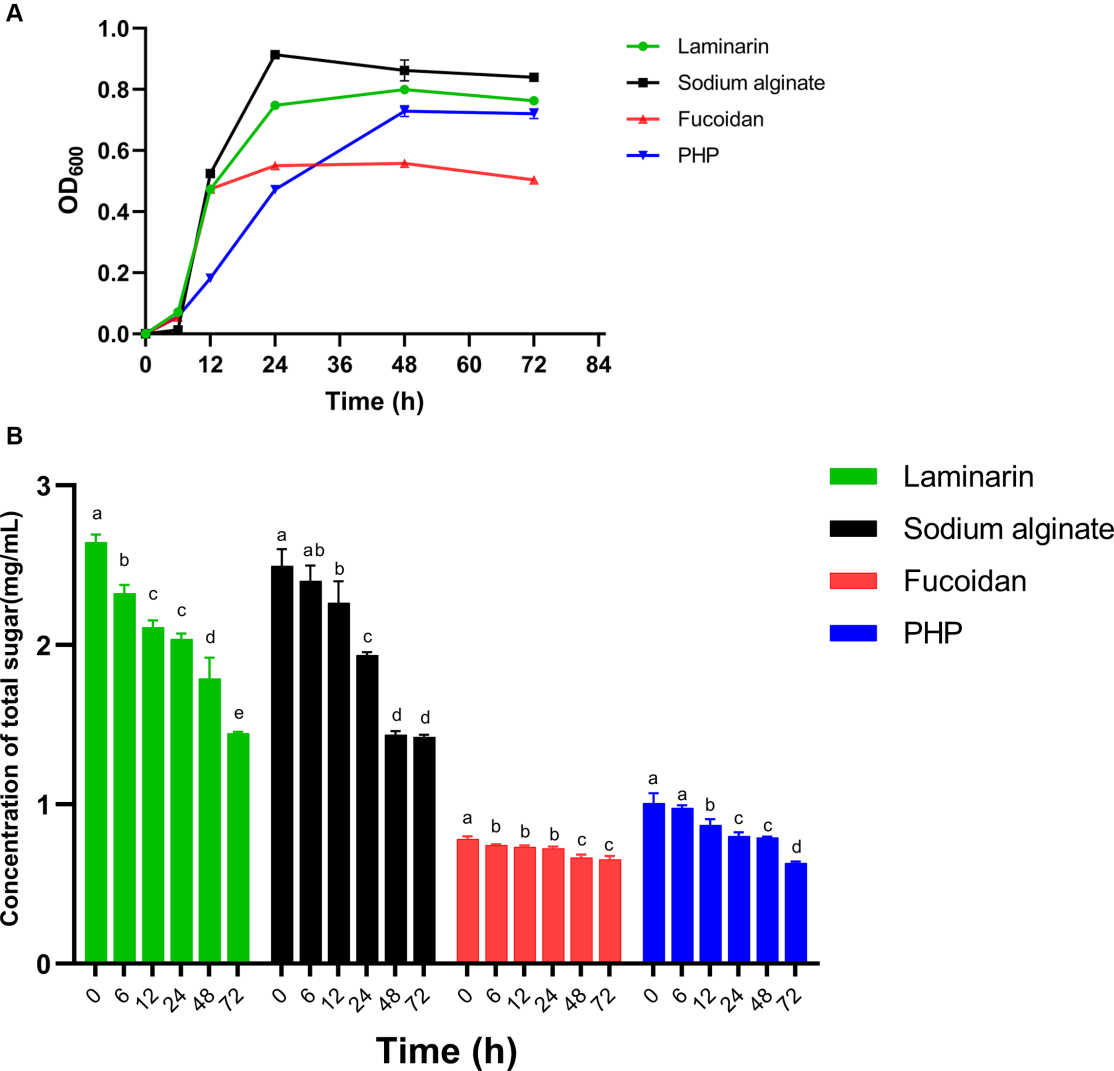
The utilization of four polysaccharides by *Bacteroides thetaiotaomicron* G4. OD600 **(A)** and sugar contents **(B)**. Different lowercase alphabet letters (a–e) are significantly different at the level of *p* < 0.05.

*Bacteroides* spp. allocate approximately 20% of their genome to metabolizing carbohydrates, whereas BT is capable of utilizing practically most polysaccharides. In turn, polysaccharides exert a beneficial impact on *Bacteroides* spp. Cultures of *B. thetaiotaomicron* G4 were cultivated in four different 0.5% algal polysaccharide mediums for 3 days, with fermentation samples obtained at 0, 6, 12, 24, 48, and 72 h to quantify the OD_600_. The growth curve (Figure 2A) showed the *B. thetaiotaomicron* could utilize these polysaccharides and substantially grow (OD_600_ > 0.5) in the basic medium containing these substates.

The strain *B. thetaiotaomicron* grew in all four polysaccharide medias, with an initial lag phase within the first 6 h, and *B. thetaiotaomicron* entered the log phase after overcoming the lag period. After roughly 24 h of cultivation, the growth of *B. thetaiotaomicron* reached its stationary phase in media containing sodium alginate, fucoidan, and laminarin. In *P. haitanensis* polysaccharide-containing medium, the maximum cell density was reached after 48 h. The cultivation in fucoidan media revealed the relatively lowest cell density, with a maximum OD_600_ value of roughly 0.5, whereas the other three cultures attained OD_600_ values surpassing 0.7.

The experimental results indicated that BT demonstrated a greater capacity for degrading sodium alginate, laminarin, and PHP, while displaying a relatively lower capability in the degradation of fucoidan. These observations were closely tied to BT’s CAZymes production capabilities and the intrinsic properties of these enzymes, which should be further investigated in the future research.

### Sugar concentration of polysaccharides residuals

The total sugar contents of bacterial culture samples, collected at different incubation times, were determined using the phenol-sulfuric acid method. Absorbance was measured at OD_490_ for the analysis of concentration changes (Figure 2B). Notably, there was a noteworthy decline in total sugar concentration in the sodium alginate, laminarin, and PHP, indicating putatively higher enzyme activity in the degradation of these polysaccharides. This suggests that *B. thetaiotaomicron* can effectively utilize these three polysaccharides, resulting in improved growth, consistent with the growth curve analysis above.

Notably, the relatively slow decline of sugar content during PHP degradation confirmed our prediction that only two PULs are involved in this process, likely attributed to lower gene expression levels associated with PHP utilization.

In the case of algal fucoidan, despite the presence of multiple polysaccharide utilization loci, there is a relatively slow decrease in carbohydrate concentration during the initial 24 h, which corresponds to the gradual increase in OD_600_. The enzyme activity may be hindered by suboptimal environmental factors, including pH and temperature, which resulted in the relatively low utilization of fucoidan and a slow strain growth rate.

### Molecular weight of polysaccharides residuals

Samples of bacterial cultures at different time points were analyzed for polysaccharide molecular weight using the HPLC-ELSD equipment. It was evident that the molecular weights of various algal polysaccharides in different carbohydrate groups had decreased (Table 1). This observation indicates that *B. thetaiotaomicron* can utilize the respective algal polysaccharides and break them down into smaller molecular substances.

**Table 1:** Changes in molecular weight (Mw) of polysaccharide.

Molecular weight of polysaccharide (Da)	Fermentation time (h)				
	0	12	24	48	72
Laminarin	23598.8	22254.13	20559.55	20319.72	18203.04
Sodium alginate	5927.23	5781.267	5333.214	5309.798	4770.658
Fucoidan	23017.67	22849.48	22123.95	20710.88	20680.52
PHP	433812.2	288126.6	201151.1	180726.9	178095.5

It is worth noting that the most substantial decrease in polysaccharide molecular weight occurs during 0–24 h, corresponding with the log phase of *B. thetaiotaomicron* growth. In the log phase, the bacteria were characterized by rapid and high metabolic activity while extensively producing and secreting CAZymes degrading polysaccharides, which was further validated by this result.

### CGView representation of genomic features of the strain

**Figure 3:**
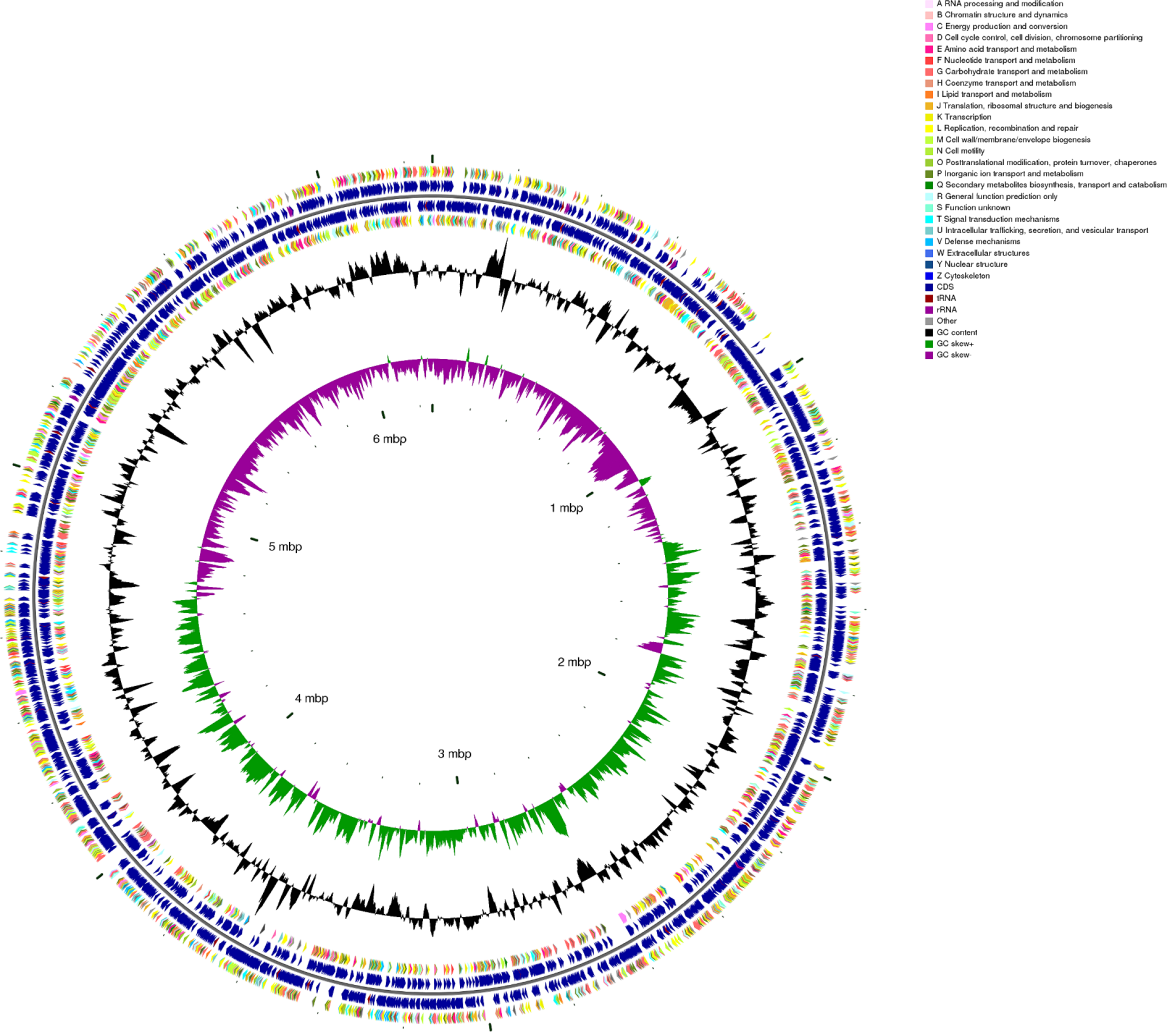
CGView map shows a full view of the genome of *Bacteroides thetaiotaomicron* G4. In the circular map, moving from the outermost ring to the innermost ring, the first and fourth rings represent coding sequences (CDS) on the forward and reverse DNA strands, with different colors denoting distinct COG (Cluster of Orthologous Groups) functional categories. The second and third rings depict CDS, tRNA, and rRNA on both the forward and reverse strands. The fifth ring displays the GC (Guanine-Cytosine) content, with outward extensions indicating regions where the GC content exceeds the genome-wide average GC content. Higher peaks in this ring correspond to greater deviations from the mean GC content, while inward extensions represent regions with GC content lower than the average, with higher peaks signifying larger differences from the mean GC content. The sixth ring represents GC-skew values, calculated using the formula (G − C)/(G + C). GC skew assists in distinguishing the leading and lagging DNA strands, with a general rule that the GC skew is greater than 0 for the leading strand and less than 0 for the lagging strand. Additionally, it can aid in identifying the replication origin (minimum cumulative offset) and termination site (maximum cumulative offset), which is particularly crucial for circular genomes. The innermost ring serves as a size indicator for the genome.

Integrating numerous forms of information into a single genome circular map helps us to acquire a more thorough and intuitive picture of the genomic properties of the bacterial strain. From the map (Figure 3), we could observe that the genes attached to carbohydrate transport and metabolism, amino acid transport, and metabolism exhibited significant expression levels. This phenomenon led to the inference that these heightened gene expressions consist of robust polysaccharide metabolism processes within *B. thetaiotaomicron* G4.

### Gene functional enrichment analysis

**Figure 4:**
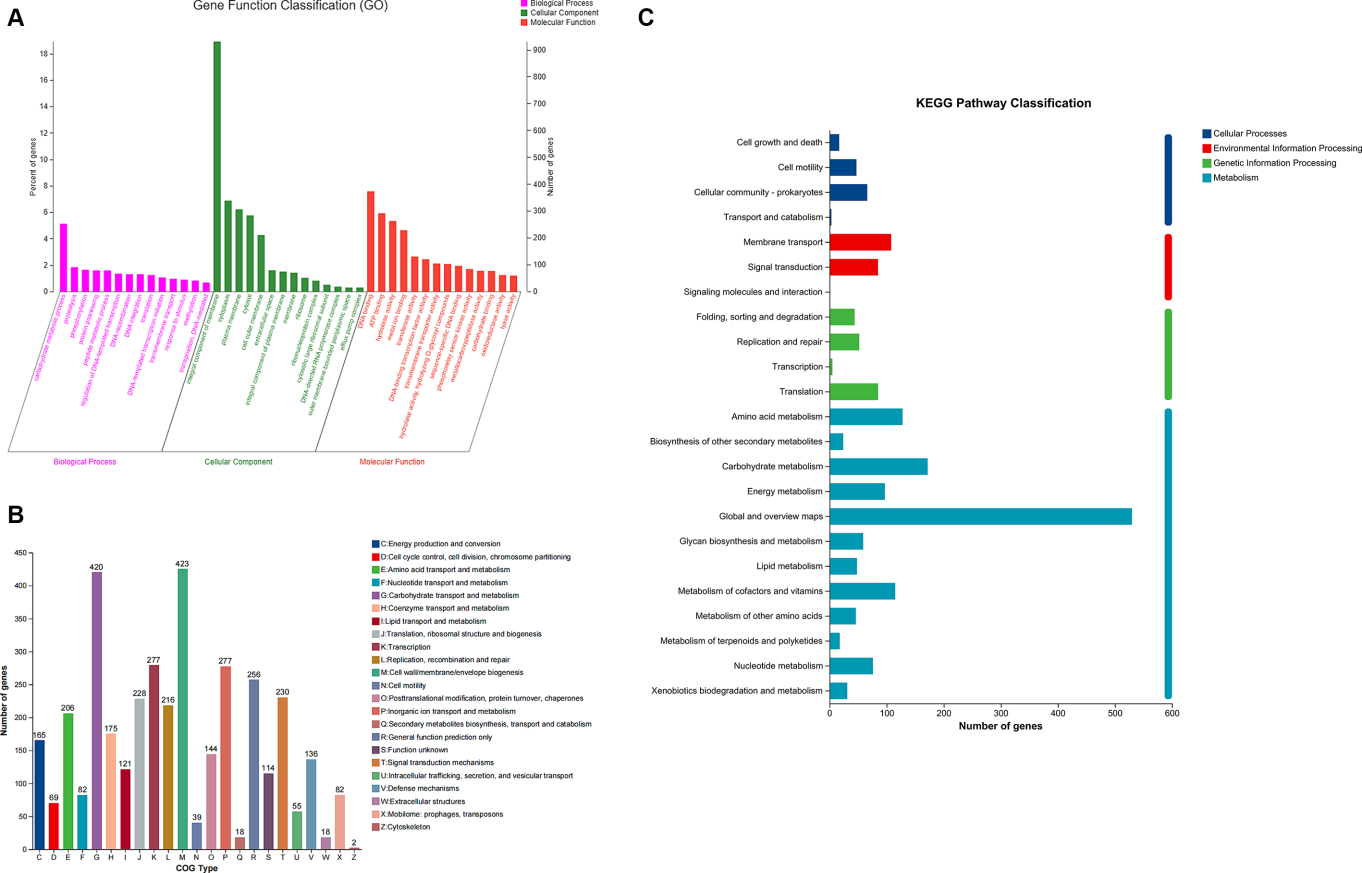
Gene ontology (GO) **(A)**, Clusters of Orthologous Groups (COG) **(B)**, and Kyoto Encyclopedia of Genes and Genomes (KEGG) **(C)** analysis of *Bacteroides thetaiotaomicron* G4.

To comprehend the biological activity of proposed genes, the genes of *B. thetaiotaomicron* G4 were annotated into three types for functional classification. The sequences in the strain’s genome were aligned with the GO (Gene Ontology) database based on sequence similarity. The results were then clustered, categorizing the genes into BP, MF, and CC categories based on their distinct functions. The top 10 subcategories of each of these three categories were further visualized in the chart (Figure 4A).

In this study, 72.54% of genes were annotated in the databases, while the rest were not. Specifically, according to the GO classification, molecular function (5,818 genes, 49.72%), cellular component (2,747 genes, 23.47%), and biological process (3,137 genes, 26.81%) were classified. Of note, the most dominant genes were enriched (253 genes, 5.14%) in the genes related to carbohydrate metabolic processes within the biological process. Besides, among the molecular functions, hydrolase activity and hydrolyzing O-glycosyl compounds were significantly enriched (103 genes, 1.77%). These results may indicate the superior potential of carbohydrate metabolism and hydrolytic activity.

### Distribution of *Bacteroides thetaiotaomicron* G4 by Cluster of Orthologous Groups annotation

By utilizing the Cluster of Orthologous Groups (COG) functional class designations (NCBI), essential genes were categorized into 23 types (Figure 4B). Besides, we recognized significant enrichment of essential gene groups, such as groups “G” (carbohydrate transport and metabolism), group “E” (amino acid transport and metabolism), and group “H” (coenzyme transport and metabolism), which were tightly linked to the degradation of polysaccharide. Specifically, there were 420 genes associated with groups “G,” 206 genes involved in groups “E,” and 175 genes participating in groups “H.” These COG groups included a variety of protein families that possess carbohydrate degradation, and facilitated the adaption of *B. thetaiotaomicron* G4 to cultural environment (Satti et al., 2018).

### KEGG pathways classification

Most of the significantly enriched GO terms in our analysis were carbohydrate metabolic processes, hydrolase activity, and hydrolyzing O-glycosyl compounds. So, the KEGG was conducted to further confirm the gene functions. The KEGG classification here (Figure 4C) was mainly enriched in the following four categories: “cellular processes” (133 genes), “metabolism” (1,344 genes), “environmental information processing” (194 genes), and “genetic information processing” (186 genes). Genes assigned to “metabolism” were distributed among 12 subgroups. The pathways within these subgroups were firmly associated with carbohydrate metabolism, energy metabolism, and glycan biosynthesis and metabolism, accounting for 12.8, 7.21, and 4.39% of all genes, respectively. These results were the same as the findings in GO and COG terms, suggesting the potential importance of *B. thetaiotaomicron* G4 in polysaccharide degradation.

### CAZymes annotations and description of the PULs dedicated to marine algae polysaccharide degradation

**Figure 5:**
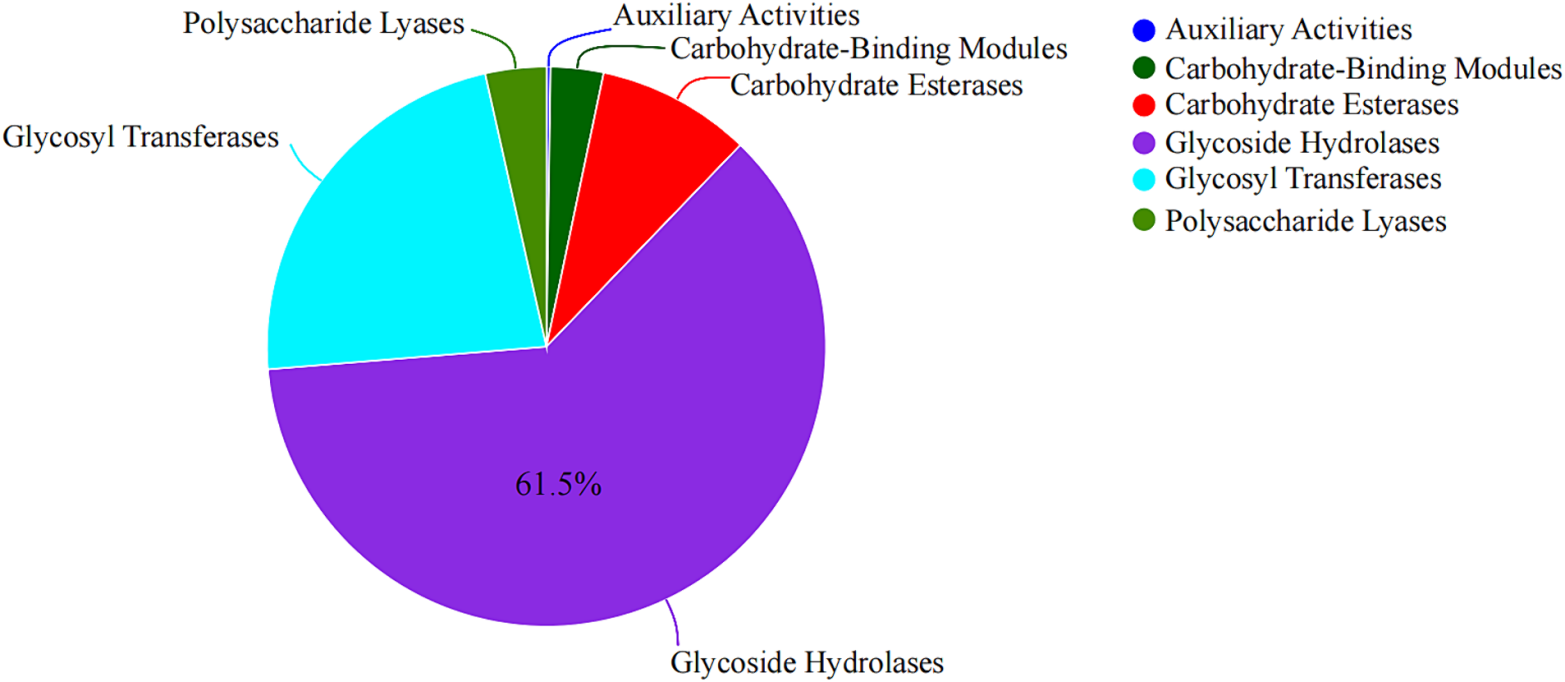
Analysis of carbohydrate active enzymes of *Bacteroides thetaiotaomicron* G4.

**Figure 6:**
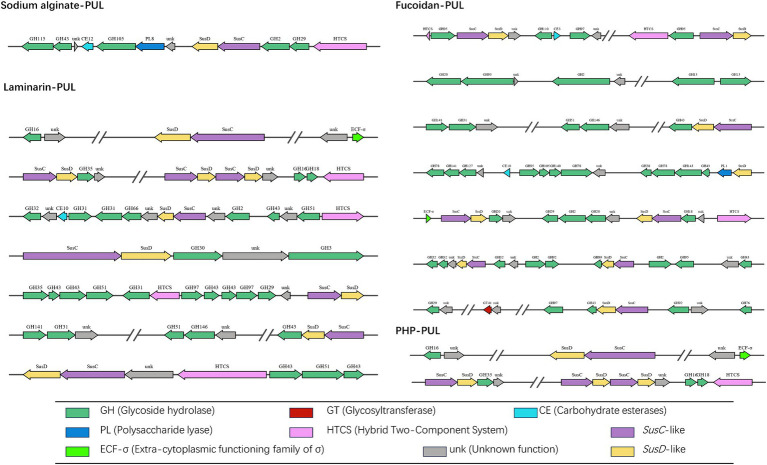
Schematic of the putative ten PULs involved in the degradation of four typical marine algae polysaccharide. The main functional modules involved in the degradation of sodium alginate were marked in bold red. *SusC*, *SusC* homolog; *SusD*, *SusD* homolog; ECF-σ, extra-cytoplasmic functioning family of *α*; HTCS, hybrid two-component system; GH, glycoside hydrolases; GTs, glycosyltransferases; CBM, carbohydrate-binding module; CE, carbohydrate esterases; PL, polysaccharide lyases; AAs, auxiliary activities; unk, unknown function.

By sequencing, we annotated the composition and proportion of CAZymes in the *B. thetaiotaomicron* G4 strain (Figure 5). One hundred twenty PULs were predicted through genomic annotation of the *B. thetaiotaomicron* G4 strain. The whole content of these *loci* was presented in Supplementary Table S1. Because of the complex arrangement of PULs, we accurately completed the division of adjacent PULs based on the previous assembly strategy (Figure 6). The cluster encoded the *SusC*/*SusD* homolog pairs and various regulatory genes, such as regulator ECF-σ and HTCS.

Sodium alginate is a water-soluble linear polymer consisting of two residues of β-(1 → 4)-D-mannuronic acid (M) and α-(1 → 4)-L-guluronic acid (G) in alternating blocks, which are irregularly arranged into homopolymers (MM blocks, GG blocks) and heteropolymers (MG blocks) (Aarstad et al., 2012). The proportions of MGs in SAs originating from different species of seaweeds are also different. The enzymes reported to hydrolyze alginate predominantly belonged to PL families, such as PL 6, PL 7, and PL 8 (Stender et al., 2019). These CAZymes functionalized through the eliminative cleavage of M/G alginate. The *B. thetaiotaomicron* G4 strain mainly contained the PL8 family M-specific alginate lyase (EC 4.2.2.3), which catalyze the degradation of alginate by a β-elimination reaction (Davidson et al., 1977). PUL 19 were predicted to be involve in the degradation of sodium alginate, and their genomic organization was depicted (Figure 6). However, the alginate lyases face much challenges in the industrial production and utilization. To address the challenges stemming from high production costs and reduced productivity, Ramya’s research team conducted screenings on samples obtained from sponges and seaweed to identify bacteria capable of producing alginate lyases. Additionally, they optimized several parameters, including the composition of the growth medium and production processes, to maximize the yield of alginate lyase (Ramya et al., 2023). Furthermore, investigations into exo-type lyases sourced from *Vibrio splendidus* have unveiled their collaborative interactions and synergistic actions in the degradation of alginate (Jagtap et al., 2014). Noteworthy was the alginate lyase, AlyC6, derived from *Vibrio* sp. NC2, which illustrated the potential for synergy between two catalytic domains (Wang et al., 2022).

Fucoidan (*U. pinnatifida* source) mainly consists of the Type I fucoidan structure, which is characterized by a linear chain of α-1,3-linked fucose residues with sulfate groups attached. The minority of the *U. pinnatifida* source fucoidan were represented by fucose and galactose residues linked by α-1,3 or alternating α-1,3/4 glycosidic bonds (Lee et al., 2004). α-L-fucosidases can hydrolytically cleave terminal α-L-fucose residues from diverse fucose-containing glycans and glycoconjugates (EC 3.2.1.51, EC 3.2.1.63, EC 3.2.1.111, and EC 3.2.1.127). Primarily found in glycoside hydrolase (GH) families 29, 95, and 141, these fucosidases act in their specific ways in different enzymatic reactions. GH29 family enzymes demonstrate versatility by cleaving α-1,2-, α-1,3-, α-1,4-, and α-1,6-linked L-fucopyranose residues from diverse glycoconjugates. In contrast, GH95 family enzymes exhibit specificity for α-1,2-linked L-fucopyranose in oligosaccharides (see text footnote 1). The enzymes from the GH141 family demonstrated specificity for L-fucose residues, specifically those replaced at 3O- with a 2-Me-α-D-Xyl residue in pectin (Ndeh et al., 2017). To further understand the feature of fucosidases, many research have been explored. For example, a comprehensive investigation was conducted, involving both bioinformatic and biochemical analyses, to explore six alpha-L-fucosidases sourced from the marine bacterium *Wenyingzhuangia fucanilytica* CZ1127(T). This study marked the first in-depth exploration of fucosidase specificity, utilizing a variety of sulfated oligosaccharides linked to fucoidan (Silchenko et al., 2022). Notably, *Lentimonas* sp. CC4 (*Verrucomicrobia*) fucoidanases have been revealed to collaborate in the degradation of fucoidan obtained from diverse brown algae sources, employing substrate-specific pathways (Sichert et al., 2020). Additionally, an investigation into the effective degradation and potential industrial applications of fucosidase involved the cloning, expression, and characterization of *Bc*FucA, a novel fucosidase originating from *Bacillus cereus* 2–8, in *E. coli* BL21 (Li Q. et al., 2021).

Laminarin is mainly β-glucans composed of β-1,3-glycosidic linkages of glucose and a small amount of β-1,6-glycosidic linkages of glucose as side chains. These endo-cleaving enzymes, known as endo-β-1,3-glucanases, can progressively cleave internal bonds within β-glucan chains (Kadam et al., 2015). Consequently, the glucan chains were internally broken into small molecular fragments. The clusters-encoded glycoside hydrolase families were predicted to be involved in the degradation of laminarin, such as GH3 (endo-1,3(4)-β-glucanase, EC 3.2.1.6), GH16 (glucan endo-1,3-β-D-glucosidase EC 3.2.1.39), and GH51 (endo-1,3(4)-β-glucanase, EC 3.2.1.6). These CZAymes could effectively catalyze the endo-hydrolysis of (1 → 3)-β-D-glucans in laminarin (Chesters and Bull, 1963; Barras and Stone, 1969). The PULs containing these families were depicted in Figure 6. Likewise, *Zg*LamC, a secreted laminarinase, is likely involved in the initial step of branched laminarin degradation, distinguishing it from the previously characterized laminarinase (Labourel et al., 2015). The expression of laminarinase activity in *Vibrio* sp. was dependent on the presence of the substrate and was repressed by the presence of glucose (Alderkamp et al., 2007). Research findings have indicated that the consumption of laminarin by *Formosa* spp. coincides with an increased uptake of diatom-derived peptides (Unfried et al., 2018).

The typical structure of *P. haitanensis* polysaccharides consists of a backbone composed of β-D-galactopyranose units linked by (1 → 3) glycosidic bonds. Additionally, the polysaccharides have two branching structures, each connected to the main chain via (1 → 4) glycosidic bonds. One branch is linked to 3,6-anhydro-α-L-galactopyranose units, and the other is linked to α-L-galactose-6-sulfate units (Zhang et al., 2004). These structural features give the polysaccharides their unique properties and functionality. During the hydrolysis process of *P. haitanensis* polysaccharides, the endo-β-1,3-galactanases (EC 3.2.1.181) and β-porphyranases (EC 3.2.1.178) assumed a pivotal role in the enzymatic degradation of PHP. Specifically, endo-β-1,3-galactanase primarily acts on the hydrolysis of β-D-galactopyranose-(1 → 4)-α-L-galactopyranose-6-sulfate linkages in porphyran, while porphyranase mainly catalyzes the hydrolysis of β-D-galactopyranose-(1 → 4)-α-L-galactopyranose-6-sulfate linkages in porphyrin (Correc et al., 2011; Kotake et al., 2011). In the present study, the data revealed that GH16 family was required to cleave the major structure of PHP, in which more than 20 kinds of enzymes could be produced. Similarly, an examination of the porphyranases’ sequences, responsible for the hydrolysis of consecutive methyl-porphyranobiose units, shed light on the diversity in the subsite specificity of porphyranases (Zhang et al., 2020).

*B. thetaiotaomicron*, a key bacterium in the human gut, shows promise in food and biotechnology. Its ability to break down complex carbohydrates offers opportunities for creating gut-friendly prebiotic foods. In biotechnology, its metabolic flexibility makes it attractive for producing bioactive compounds, biofuels, and biodegradable plastics through genetic manipulation (Liu et al., 2021; Béchon et al., 2022; Meslé et al., 2023). Additionally, it can modulate the immune system and reduce colonic inflammation, suggesting potential for treating conditions like inflammatory bowel disease (Kim et al., 2021; Li K. et al., 2021). Research is active in enhancing its probiotic traits and immune-regulating abilities, which could lead to innovative therapies leveraging the gut microbiome for better health outcomes. Overall, *B. thetaiotaomicron* holds vast potential for improving human health and sustainable biotechnology.

## Conclusion

Marine algal polysaccharides, such as sodium alginate, fucoidan, laminarin, and *Pyropia haitanensis* polysaccharide, manifest remarkable attributes that underscore their essential role across diverse applications, ranging from biotechnology to healthcare. Furthermore, the pivotal role of CAZymes in polysaccharide degradation is of utmost importance for comprehending the intricate relationship between CAZymes and marine algal polysaccharides. In this study, we delved into the underlying mechanisms of polysaccharide degradation by *B. thetaiotaomicron* G4, focusing on four typical marine algae polysaccharides: sodium alginate, fucoidan, laminarin, and *Pyropia haitanensis* polysaccharides. Pure culture experiments with *B. thetaiotaomicron* G4 in a medium where these polysaccharides served as the carbon source were conducted. The measurement of growth curve of *B. thetaiotaomicron* G4, total carbohydrate content, and molecular weight of polysaccharides residuals confirmed the utilization and degradation of polysaccharides. To further validate our analyses, we employed several annotations, such as GO annotation, COG annotation, and exploration of the KEGG pathway enrichment to identify potential target genes and pathways. Our findings revealed several PULs that were likely involved in the degradation of these four polysaccharides. This comprehensive exploration of the mechanisms behind polysaccharide degradation by *B. thetaiotaomicron* G4 sheds light on the intricate interplay between marine seaweed polysaccharides and gut microbes. In forthcoming research, the anticipated PULs identified within this investigation will undergo further examination to elucidate the intricate process of polysaccharide degradation by *B. thetaiotaomicron*. These investigations may not only expand our knowledge of enzyme specificity but also hold promise for innovative applications in biotechnology, environmental sustainability, and pharmaceutical development.

## Data availability statement

The datasets presented in this study can be found in online repositories. The names of the repository/repositories and accession number(s) can be found at: https://www.ncbi.nlm.nih.gov/, SRR26628481.

## Author contributions

BY: Data curation, Investigation, Methodology, Writing – original draft. ZL: Methodology, Validation, Writing – review & editing. SZ: Conceptualization, Funding acquisition, Methodology, Supervision, Writing – review & editing. K-LC: Conceptualization, Methodology, Supervision, Writing – review & editing.

## Footnotes

^1^ http://www.cazy.org/

^2^ http://geneontology.org/

^3^ https://www.kegg.jp/

^4^ http://bcb.unl.edu/dbCAN2/

^5^ http://pfam.xfam.org/

^6^ http://www.cazy.org/PULDB/
