# Supplementary material for: Exploring potential polysaccharide utilization loci involved in the degradation of typical marine seaweed polysaccharides by Bacteroides thetaiotaomicron
Source: Front Microbiol. 2024 May 9;15:1332105. doi: 10.3389/fmicb.2024.1332105 (PMC11119289; doi:10.3389/fmicb.2024.1332105)
Supplement: Supplementary file 1 [file Table_1.DOCX]

**Supplementary Material**

**Table S1.** The genomic annotation of each PUL

| PULs number | genomic annotation |
| --- | --- |
| PUL1 | -unk-GH77-unk-GT4-GT2- |
| PUL2 | -GH2-GH2-GH29-unk-SusD-SusC-unk-ECF-σ-GH92-unk-SusD-SusC- |
| PUL3 | -GT41-PL10-unk-SusD-SusC-SusC-SusD-unk-SusC-SusD- |
| PUL4 | -unk-unk-SusC-SusD- |
| PUL5 | -ECF-σ-unk-SusC-HTCS-unk-SusC-SusD- |
| PUL6 | -unk-GH20-SusD-SusC- |
| PUL7 | -GH78-GH78-CE7-unk-SusC-SusD- |
| PUL8 | -GH16-unk-SusD-SusC-unk-ECF-σ- |
| PUL9 | -GH97-CE12-GH67-GH76-unk-SusD-SusC-unk-HTCS-GH92-GH76-GH125 |
| PUL10 | -GH27-GT41-unk-SusC- |
| PUL11 | -unk-GH2-unk-CE3- |
| PUL12 | -unk-GT30-unk-GH123- |
| PUL13 | -SusC-SusD-GH35-unk-SusC-SusD-SusC-SusD-unk-GH16-GH18-HTCS- |
| PUL14 | -GT41-unk-GH36-GH43-GH2-SusD-SusC-HTCS-unk-GT4-GT4-GT4- |
| PUL15 | -GT2-unk-GT14-GT14-unk-GT8-GT2-GT2-unk-GT2- |
| PUL16 | -GH93-SusD-GH43-HTCS-GH43-unk-GH43-GH159-SusD-SusC-GH99-GH99-GH116-unk-GH146-GH106-GH88- |
| PUL17 | -GH154-unk-SusD-SusC-GH105-GH2-HTCS- |
| PUL18 | -GT4-GT4-GT4-unk-GT4-GT2-GH92-GH76-unk-SusD-SusC- |
| PUL19 | -GH115-GH43-unk-CE12-GH105-PL8-unk-SusD-SusC-GH2-GH29- HTCS- |
| PUL20 | -SusC-SusD-unk-SusC-GH154-SusC-SusD-GH30-unk-GH130- |
| PUL21 | -GH30-unk-SusD-SusC-unk-HTCS-GH18-unk-GH27- |
| PUL22 | -GH32-unk-CE10-GH31-GH31-GH66-unk-SusD-SusC-unk-GH2-GH43-unk-GH51-HTCS- |
| PUL23 | -CE10-SusD-SusC-unk-GH43-CE1-GH2-unk-GH92-GH36-HTCS- |
| PUL24 | -HTCS-GH95-SusC-SusD-unk-GH110-CE3-GH97-unk-HTCS-GH95-SusC-SusD- |
| PUL25 | -unk-GH20-GH2-unk- |
| PUL26 | -SusD-SusC-SusC-SusD-unk-CE4- |
| PUL27 | -ECF-σ-unk-SusC-SusD- |
| PUL28 | -ECF-σ-unk-SusC-SusD-unk-CE1- |
| PUL29 | -GH2-GH2-GH97-unk-SusD-unk-GH31-unk-GH76-HTCS- |
| PUL30 | -SusC-SusD-GH30-unk-GH3- |
| PUL31 | -PL29-unk-SusD-SusC-HTCS-unk-GH2-unk-SusD-unk-GH88- |
| PUL32 | -GT9-GT90-unk-GT2-GT4-GT4-GT4-GT2-GT2-GT2-unk-GT14-GT2- |
| PUL33 | -unk-CE4-GT87-GT87- |
| PUL34 | -GT28-ECF-σ-HTCS-GH43-unk-SusD-SusC-GH76-SusD- |
| PUL35 | -unk-SusD-SusC- |
| PUL36 | -SusC-SusD-unk-GT41-unk-GH2-GH115-GH43-GH43- |
| PUL37 | -SusD-GH76-unk-unk-GH76-unk-unk-GH92-GH125-GH92-GH127- |
| PUL38 | -GH144-unk-SusD-SusC-unk-unk-GT66- |
| PUL39 | -CE9-GH89-GH27-GH129-unk-GH20-GH2-unk-SusD-SusC-unk-CE15- |
| PUL40 | -GH35-GH43-GH43-GH51-GH31-HTCS-GH97-GH43-GH43-GH97-GH29-unk-SusC-SusD- |
| PUL41 | -SusC-GH127-GH43-unk-GH154-HTCS-SusC-SusD-unk-GH43-GH43-GH145-GH105- |
| PUL42 | -GT2-unk-unk-GH13-SusD-SusC-GH97-GH13- |
| PUL43 | -HTCS-unk-unk-unk-SusC-SusD-GH18- |
| PUL44 | -GH92-GT32-GT32-unk-GH130-GH125-GH76-GH92-HTCS-SusC-SusD-GH76-unk-unk-GH29-HTCS- |
| PUL45 | -SusC-SusD-unk-GH92-unk-GH99-unk-GH20- |
| PUL46 | -unk-unk-SusC-unk-SusC- |
| PUL47 | -GT41-unk-unk-HTCS-SusC-SusD-unk-GH29-HTCS-SusC-SusD-unk-GH92-GH92-unk-GH92- |
| PUL48 | -SusD-unk-unk-GH92-GH92-ECF-σ-GH92-unk-GH23-unk-GH19- |
| PUL49 | -SusD-SusC-unk-unk-GH2-unk-SusC- |
| PUL50 | -unk-GH92-GH2-CE3-GH78-CE1-CE3-unk-unk-SusC-SusD- |
| PUL51 | -SusD-unk-unk-SusC-SusD-SusC-unk-GH92-GH92-GH130-GH43-CE3- |
| PUL52 | -GH105-CE8-CE8-HTCS-unk-SusD-SusC-PL1-PL1-unk-unk-PL1-SusD-GH28-HTCS- |
| PUL53 | -SusD-unk-GH29-HTCS- |
| PUL54 | -GH106-GH28-unk-GH28-CE12-GH2-unk-GH42-GH28-CE4-GH28-GH2-unk-CE12-unk-GH35- |
| PUL55 | -PL9-unk-PL26-CE12-GH105-PL11-GH105-unk-HTCS-CE6-GH2-HTCS-PL9-unk-GH43-unk-GH28- |
| PUL56 | -HTCS-unk-GH2-unk-SusD-unk-ECF-σ-GH110- |
| PUL57 | -GT2-GT4-unk-unk-unk-SusD-unk-unk-GH133-GT4-GH57-unk-GT5- |
| PUL58 | -unk-SusD-GH89-unk- |
| PUL59 | -unk-GH20-GH84-unk-unk-ECF-σ- |
| PUL60 | -unk-SusC-SusD- |
| PUL61 | -SusD-SusC-unk-unk-ECF-σ- |
| PUL62 | -unk-GH88-SusD-SusC- |
| PUL63 | -unk-HTCS-unk-GH2-GH53-unk-SusD-SusC-GH147-HTCS-unk-PL13- |
| PUL64 | -GH20-GH95-unk-GH2-unk-GH13-GH13- |
| PUL65 | -SusC-SusD-GH18-unk-unk-GH29- |
| PUL66 | -ECF-σ-unk-SusC-SusD-unk-SusC-SusD-unk-unk-GT2-GT2-GT2-GT2-GT101-GT2-GT2-GT4-GT4-GT4- |
| PUL67 | -GH97-unk-GH146-HTCS-unk-SusC-SusD-unk-GH43-GH88-unk-SusC-unk-CE1- |
| PUL68 | -ECF-σ-SusC-SusD-SusC-SusD-GT2-PL27-PL27-GH43-HTCS-SusD-SusD-GH35- |
| PUL69 | -GH25-unk-unk-SusC-SusD-unk-GH109- |
| PUL70 | -GH141-GH31-unk-GH51-GH146-unk-GH43-SusD-SusC- |
| PUL71 | -SusD-SusC-unk-HTCS-GH43-GH51-GH43- |
| PUL72 | -GT4-GT4-unk-GT4-unk-unk-unk-GT41-unk-unk-GH76-SusC-SusD-unk-CE2- |
| PUL73 | -SusD-SusC-GH89-unk-GH33-GH20-unk-GH2-GH20-GH20-GH2-unk- |
| PUL74 | -unk-GT2-unk-GT2-unk-SusC-SusD-unk-unk-SusC- |
| PUL75 | -unk-SusC-unk-SusC-unk-GH20- |
| PUL76 | -CE3-unk-unk-CE1-unk-unk-GT4-unk-GT94-GT4- |
| PUL77 | -unk-CE9-CE9-unk-unk-unk-GH97- |
| PUL78 | -SusC-SusD-unk-unk-GH2-unk-unk-GT41-unk-unk- unk-GH13-unk-GH13- |
| PUL79 | -unk-SusD-SusC-unk-unk-unk-GT41-unk-unk-GT41- |
| PUL80 | -HTCS-SusC-unk-GT41-unk-PL1-HTCS-GH2-GH139-GH106-unk-GH2-GH2-unk-GH2-GH138- |
| PUL81 | -GH78-GH141-GH127-unk-CE10-GH95-GH105-GH140-GH78-unk-GH28-GH78-GH143-GH43-unk-PL1-SusD- |
| PUL82 | -SusD-unk-GH92-GH130-GH163-unk-SusD-SusC-SusC-SusD-GH18-unk-GH20-unk-SusC- |
| PUL83 | -CE10-unk-SusC-unk-GT35-unk-unk-SusD-SusC- |
| PUL84 | -unk-GT2-GT2-unk-GT2-GT101-GT4-GT2-unk-CE10-GT51-unk-ECF-σ- |
| PUL85 | -CE1-unk-unk-ECF-σ-unk-SusD-unk-GH18-unk-GT35-GT3- |
| PUL86 | -GT4-unk-GT2-GT4-unk-GT2-unk-GT4-GT2- |
| PUL87 | -unk-unk-SusD-SusC- |
| PUL88 | -unk-SusC-SusD-unk-unk-ECF-σ- |
| PUL89 | -ECF-σ-SusC-SusD-GH20-unk-GH29-GH2-GH20-unk-SusD-SusC-GH18-unk-HTCS- |
| PUL90 | -GT2-GT2-unk-GT2-GT25-unk- |
| PUL91 | -SusD-SusC-unk-GT41-unk-unk-GT2-GT4-unk-unk-ECF-σ-unk-HTCS- |
| PUL92 | -GH32-GH32-unk-SusD-SusC-GH32-unk-GH2-GH92-GH99-SusD-SusC-unk-GH2-GH95-unk-GH43- |
| PUL93 | -GH29-unk-GT41-unk-unk-GH97-GH43-SusD-SusC-GH92-unk-GH76- |
| PUL94 | -unk-SusC-SusD-unk- |
| PUL95 | -GH92-unk-SusC-SusD-unk-unk-GH92-GH43- |
